# Family physician involvement in aesthetic medicine in British Columbia: a cross sectional environmental scan

**DOI:** 10.1186/s12875-026-03301-w

**Published:** 2026-04-11

**Authors:** Borum Yang, Olivia Gemmill, Julie Easley, François Gallant, Erin Palmer, Ruth Lavergne

**Affiliations:** 1 Department of Family and Community Medicine, University of Toronto, Toronto, ON Canada; 2 Faculty of Medicine, Dalhousie University, Halifax, NS Canada; 3 Faculty of Science, Dalhousie University, Halifax, NS Canada; 4 Department of Medical Education, Horizon Health Network, Fredericton, NB Canada; 5 Department of Family Medicine, Dalhousie University, Halifax, NS Canada; 6 Vitalité Health Network, Bathurst, NB Canada; 7 Faculty of Medicine, Dalhousie University, Halifax, NS Canada

**Keywords:** Private healthcare sector, Aesthetic medicine, Primary care workforce, Healthcare workforce, Canada, Environmental scan

## Abstract

**Background:**

Aesthetic medicine is a rapidly expanding area of practice that operates outside the publicly funded healthcare system in Canada. As a result, physician involvement in this sector is not captured in administrative data sets and the extent of family physician participation remains poorly characterized. We conducted an environmental scan of the publicly observable footprint of aesthetic medicine in British Columbia (BC), including clinics advertising minimally invasive procedures, and the physicians publicly associated with them with specific attention to the representation of family physicians.

**Methods:**

Clinics were identified using Allergan Canada’s public clinic locator and supplemented with an independent Google search. Clinics offering onabotulinumtoxin A and hyaluronic acid dermal fillers were included. Physicians affiliated with included clinics were verified through the College of Physicians and Surgeons of British Columbia (CPSBC) registry. Descriptive analyses were performed to characterize clinics and physicians including specialty distribution, training background, and clinic staffing.

**Results:**

A total of 171 clinics and 299 physicians were identified. Family physicians represented the largest specialty group (192/299, 64.2%), followed by dermatology (13.7%) and plastic surgery (10.0%). Most providers were Canadian trained (67.6%), and gender distribution was balanced (53.5% male, 46.5% female). Forty-eight physicians (16.1%) reported additional training, and nine practiced across multiple sites. Clinics were concentrated in the Mainland/Southwest region (62.0%), with over half employing allied health professionals, most commonly registered nurses. Ownership information was reported for 21.0% of clinics, and documentation of prescriber oversight was inconsistent.

**Conclusion:**

This study provides a conservative estimate of aesthetic medicine activity in BC and identifies family medicine as the most represented specialty among publicly listed providers. These findings highlight the need for more comprehensive data on privately paid medical services to better understand practice models and how aesthetic medicine fits within Canada’s evolving primary care landscape.

## Background

The medical aesthetics industry is expanding rapidly, with growing participation from multiple regulated health professions in the provision of minimally invasive procedures, including registered nurses, nurse practitioners, and physicians [1, 2]. In studies from the United States (US), family medicine has been observed as one of the fastest-growing provider segments in this field [3, 4]. Whether similar patterns exist in Canada remains unclear.

At the same time, access to primary care remains an urgent challenge in Canada despite increasing per-capita supply of family physicians and efforts to expand family medicine residency programs [5, 6]. Evidence suggests that family physicians are increasingly taking on focused practice roles such as working in emergency departments, as hospitalists, among others, with the Canadian Institute for Health Information (CIHI) reporting nearly 30% of family physicians in Canada now delivering services outside of comprehensive primary care [7]. However, these figures only account for services that can be tracked through public billing and exclude privately paid services, such as aesthetic medicine.

Family physician involvement in aesthetic medicine in Canada is therefore poorly characterized. A recent environmental scan identified clinics across several provinces with 273 family physicians offering privately paid services, but no specific data on aesthetic medicine were collected [8]. Moreover, aesthetic medicine operates outside the publicly funded system, and there are no centralized data sets or registries that systematically track clinics or providers engaged in this area of practice. As a result, the scope and distribution of physician involvement in aesthetic medicine remains largely invisible within conventional health workforce data. This lack of visibility is consistent with broader observations that privately-funded health care is insufficiently captured within routine health system data, despite potential implications for workforce planning and system governance [9]. Similarly, understanding the extent of family physician involvement in aesthetic medicine would be important for evaluating potential implications for the primary care workforce and service availability.

In this context, environmental scans of publicly available information provide a pragmatic approach to generating baseline descriptive data in emerging or poorly captured areas of healthcare. Accordingly, we conducted a cross-sectional environmental scan to describe the publicly available footprint of aesthetic medicine in British Columbia (BC), including clinics advertising injectable services and the physicians publicly associated with them, with specific attention to the representation of family physicians. BC was selected as the setting because its large, diverse, and urbanized population, along with above-average disposable income, are factors associated with higher demand for aesthetic procedures, making it a suitable setting to examine this practice landscape [10].

## Methods

### Study design

We conducted a cross-sectional environmental scan to identify clinics in BC that publicly advertise minimally invasive aesthetic procedures, specifically neuromodulator (onabotulinumtoxin A) and hyaluronic acid-based dermal fillers. These were selected because they represent the most commonly performed minimally invasive aesthetic procedures globally and account for the majority of injectable aesthetic treatments in practice [11].

### Sampling and clinic identification

Clinics were identified using Allergan/Abbvie public clinic locator, a directory of practices that offer their products (BOTOX^®^ and JUVÉDERM^®^) [12]. Allergan/AbbVie products represent the largest share of neuromodulators and fillers globally and within Canada, and peer reviewed publications and contemporary market analyses show that these agents are widely stocked across aesthetic practices, even when alternative brands are also used [13–15]. The locator therefore served as a practical initial sampling frame. To improve completeness, we supplemented the locator with independent Google search using the following terms: “botox clinic British Colombia”, “dermal fillers British Columbia”, “cosmetic clinics British Columbia”, “aesthetic clinics British Columbia”.

### Inclusion and exclusion criteria

Clinics were excluded if they: provided onabotulinumtoxin A for non-cosmetic indications (e.g., chronic migraine, dystonia) in publicly funded settings; lacked publicly accessible website (because services offered and staffing structures cannot be verified); or did not list a physician (medical doctor [MD]) who could be cross-referenced through CPSBC registry. These criteria ensured standardized verification of clinic type, service scope and physician identity.

### Data extraction

For each clinic website, we collected information on the MDs listed, presence of allied health professionals (e.g., nurse practitioners, registered nurses, licensed practical nurses), non-regulated staff (e.g., aestheticians, laser technicians) which were coded as binary variables (yes/no) based on explicit listing on clinic websites. To characterize clinic governance transparency, we documented whether the clinic website explicitly identified a regulated health professional authorized to prescribe the injectable agents and oversee clinical practice (hereafter referred to as “prescriber oversight”). We collected information on geographic location based on BC Health Authorities. Additional observations relevant to clinic structure or staffing were recorded descriptively.

### Physician identification and verification

For every physician identified, credentials including board certification, location of training (Canada/international), year of MD, and gender were collected from the CPSBC public registrant directory [16]. All clinic searches, website data extraction and CPSBC verification were conducted between September 1, 2024, and October 31, 2024. Two reviewers (BY, OG) independently extracted and verified data. Discrepancies were resolved through discussion with senior investigators (RL, JE).

These outcomes were pre-defined to describe the scope of family physician involvement in aesthetic medicine and to contextualize potential implications for the broader primary care workforce.

### Analysis

Data were summarized using descriptive statistics only, including frequencies and percentages. Physician-level characteristics were stratified by specialty.

## Results

### Study population

**Fig. 1:**
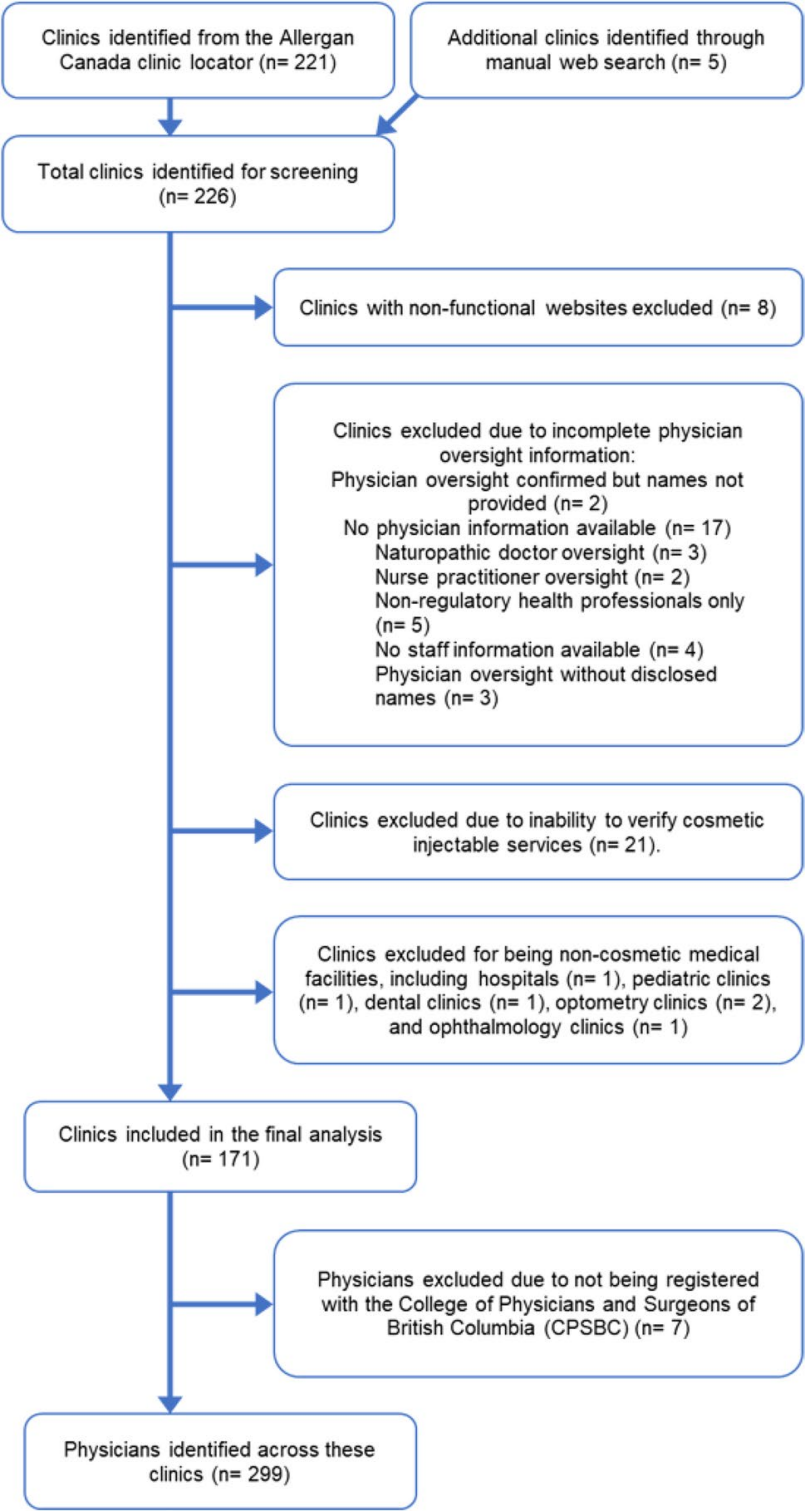
Flow diagram of the clinic and physician selection process

We identified 226 clinics in total, with 221 sourced from the Allergan website and 5 from a manual Google search. After applying exclusion criteria, 171 clinics and 299 physicians were included in the final analysis. A total of 55 clinics were excluded due to lack of publicly available websites, no confirmation of offering cosmetic Onabotulinumtoxin A and filler services, or no public listing of an associated licensed medical doctor (MD). (Fig. 1). Additionally, 7 physicians were excluded due to lack of active registration with theCPSBC.

### Physician characteristics

**Fig. 2:**
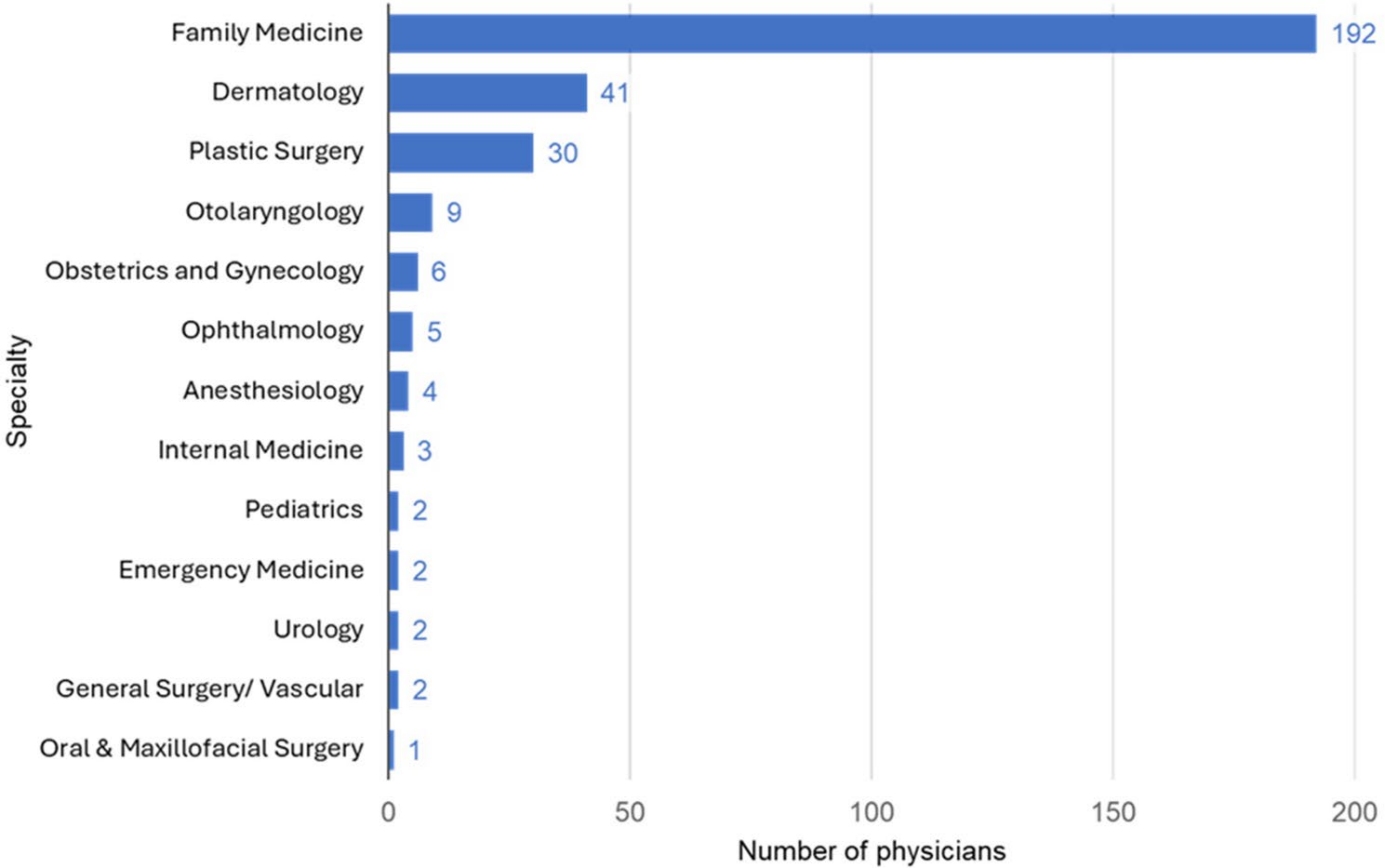
Specialty training of physicians publicly affiliated with aesthetic medicine clinics in British Columbia. *A small number of physicians also had combinations, including family medicine and plastic surgery (3), dermatology and plastic surgery (1), dermatology and family medicine (4), otolaryngology and family medicine (3), family medicine and ophthalmology (3), or other combinations (5)

Among the 299 physicians, 13 medical specialties were represented (Fig. 2). Family physicians constituted the largest group, with 192 physicians (64.2%) practicing across 116 clinics (Table 1). Dermatology followed with 41 physicians (13.7%), and plastic surgery with 30 physicians (10.0%). Physician titles commonly included roles such as Medical Director, Cosmetic Injector, Board-Certified Esthetic Physician, and Cosmetic Doctor. Nine physicians (3%) were listed on more than one included clinics, ranging from 2 to 4 clinics.

**Table 1:** Demographic, training and career-stage characteristics of physicians publicly affiliated with aesthetic medicine clinics in British Columbia

Characteristic	All physicians (*n* = 299)	Family Medicine (*n* = 192)	Dermatology (*n* = 41)	Plastic Surgery (*n* = 30)	Other Specialties* (*n* = 36)
Total number of physicians	299	192	41	30	36
Sex, n (%)					
Female	139 (46.5)	91 (47.4)	27 (65.9)	9 (30.0)	12 (33.3)
Male	160 (53.5)	101 (52.6)	14 (34.1)	21 (70.0)	24 (66,7)
Training Background, n (%)					
CMG	202 (67.6)	102 (53.1)	40 (97.6)	29 (96.7)	31 (86.1)
IMG	97 (32.4)	90 (46.9)	1 (2.4)	1 (3.3)	5 (13.9)
Years since MD graduation, n (%)					
≤ 10 years	–	19 (9.9)	–	–	–
11–20 years	–	68 (35.4)	–	–	–
> 20 years	–	105 (54.7)	–	–	–

n indicates the total count in each column subgroup; values are presented as n (%), where the percentage is calculated out of that subgroup’s n. Years since MD graduation was calculated for family physicians only

*CMG* Canadian medical graduates, *IMG* international medical graduates

*Other specialties include otolaryngology, ophthalmology, anesthesiology, emergency Medicine, general/vascular surgery, internal medicine, urology, pediatrics, and oral & maxillofacial surgery

Regarding medical training, 202 providers (67.6%) were Canadian-trained, while 97 (32.4%) were international medical graduates (IMGs) (Table 1). Some physicians (*n* = 48; 16.1%) reported having additional training in cosmetic dermatology or related fields on clinic websites, including postgraduate diplomas in dermatology and certifications from aesthetic and cosmetic organizations. As of 2025, 105 (54.7%) family physicians had graduated more than 20 years prior, 68 (35.4%) between 11 and 20 years, and 19 (9.9%) within the past 10 years. The gender distribution was balanced, with 53.5% male and 46.5% female providers.

### Clinic characteristics

Of the 171 clinics, 19 (11%) listed teams including more than one physician specialty. The most common combination was Family Medicine and Dermatology (4 clinics), followed by Family Medicine and Plastic Surgery (3 clinics). Ownership information was available for 36 clinics (21.0%), and 70 physicians (23.4%) explicitly stated they personally performed cosmetic injections (Table 2). Clinics were unevenly distributed across the province, with the majority located in the Mainland/Southwest (62.0%), followed by Vancouver Island/Coast (18.7%) and Thompson-Okanagan (13.5%) (Table 2).

**Table 2:** Characteristics of publicly identified clinics offering aesthetic medical services in British Columbia

Clinic characteristic	Number of clinics (*n* = 171)	% of clinics
Clinic staff characteristics		
Non-registered professional present	71	41.5
Allied healthcare professional present	91	53.2
Physician owner	36	21.0
Physician-administered injections	71	23.0
Multi-specialty physician teams	19	11.0
Clinic location (economic region*)		
Mainland/Southwest	106	62
Vancouver Island/Coast	32	18.7
Thompson-Okanagan	23	13.5
Kootenay	5	2.9
Cariboo	3	1.8
Northern (Nechako, North Coast, Northeast)	2	1.2
Nechako	1	0.6
North Coast	1	0.6
Northeast	0	0

n indicates the total count of clinics. Percentages are calculated out of total clinics (*n* = 171)

*Economic regions as defined by Statistics Canada’s 2006 Standard Geographical Classification (SGC): Mainland / Southwest (Vancouver Coastal + Fraser Valley), Vancouver Island / Coast (Vancouver Island + Sunshine Coast), Thompson – Okanagan (Thompson Nicola + Okanagan), Kootenay (East + West Kootenay), Cariboo, Northern region (comprising Nechako, North Coast and Northeast)

Allied healthcare professionals were identified in 91 clinics (53.2%). These included 9 nurse practitioners, 107 registered nurses, 25 licensed practical nurses, 8 naturopathic doctors (a regulated health profession in BC), and 2 physician assistants. The average was 1.7 (standard deviation 2.1) allied professionals per clinic. Additionally, 71 clinics (41.5%) employed non-regulated professionals such as aestheticians, laser technicians, and aesthetic consultants.

## Discussion

This environmental scan offers an initial overview of clinics and physicians publicly advertising aesthetic injectable services in BC. In the absence of a centralized registry or comprehensive dataset for this sector, our findings serve as a preliminary yet informative estimate in a field where population-level data is currently lacking. Given the reliance on publicly available information, it is likely that the number of clinics and providers captured here is underrepresented. Nevertheless, this work provides initial understanding of the scale and nature of aesthetic medical services and highlights important knowledge gaps that need more robust data to address.

Across 171 clinics, we identified 299 physicians involved in aesthetic services in BC, with family physicians comprising the largest specialty group. Based on CIHI 2023 workforce data, this represents approximately 2.5% of all family physicians in the province [17]. This proportion should be interpreted carefully, as it reflects only those physicians publicly listed on clinic websites in our sampling frame. The extent to which these providers participate in aesthetic services full time, part time, or alongside longitudinal primary care cannot be determined as publicly available information does not reliably capture other practice locations or time spent across settings. Nonetheless, the finding that family physicians represent a large share of publicly listed providers prompts questions about how aesthetic medicine is incorporated into their broader practice patterns, something worth exploring in future studies. Family physicians involved in aesthetic medicine were not typically early in practice, despite some speculation that early-career physicians might be more commonly involved in focused practice areas [18].

In addition to workforce trends, our findings also highlight significant gaps in how clinics document medical oversight. Only one-fifth of clinics reported ownership information, and documentation of authorized prescriber was inconsistent. These findings mirror previous work showing inconsistent oversight in medical spas, where supervising physicians were absent in 81% of facilities and only 64% of patients were informed of the supervising physician’s identity [19]. While website-based data cannot confirm actual supervision practices, the inconsistency in publicly reported information raises important concerns about the adequacy of clinical oversight in aesthetic medicine, particularly in settings where non-physician providers perform procedures. Reporting of additional training was also variable across clinic websites, and it is unclear whether the observed proportion reflects absence of training or inconsistent reporting. As the field continues to grow, greater transparency around prescriber roles and training may be important, particularly given that most current training is offered through private, non-accredited programs rather than formal medical education pathways [20].

Previous work has shown that aesthetic procedures are performed by aestheticians and nurses, with nurse injectors outnumbering physicians in many settings [19]. In contrast, our data identified 299 physicians compared to 151 non-physician professionals. However, that information on non-physician professionals was only available for 53.2% of the clinics. Therefore, the discrepancy likely reflects incomplete website reporting rather than true differences in who is performing procedures.

To our knowledge, no previous study has attempted to quantify the involvement of family physician participation in aesthetic medicine in Canada, making these findings an initial step towards better understanding this sector. An important methodological limitation of this study is the use of the Allergan clinic locator as the primary sampling frame. While Allergan produces the most widely recognized neuromodulator brand which makes it a practical starting point in the absence of a central registry, this approach does not capture clinics that exclusively use other manufacturers such as Galderma. Additionally, clinics may also hold accounts with multiple manufacturers while choosing to affiliate publicly with only one. For these reasons, our estimates likely exclude a portion of clinics and providers active in this space. Additional limitations relate to the nature of website based data. Websites vary in completeness, accuracy and update frequency, and not all clinics list all staff. In total, 55 clinics were excluded from our analysis due to insufficient or missing website information. As such, online presence should not be assumed to accurately reflect actual clinical activity or staffing patterns. Lastly, the observed specialty distribution should be interpreted with caution, as our approach may not capture dermatologists or plastic surgeons who provide cosmetic procedures within general practice settings without marketing aesthetic services as a distinct area of care.

Environmental scans are widely used to map emerging areas of healthcare when centralized data sources are lacking. In the case of aesthetic medicine, which largely operates outside of the public billing system and is therefore invisible in administrative data sets, publicly available information provides one of the few viable tools for generating baseline descriptive data. As such, these findings offer a reference point while also highlighting the need for improved mechanisms to track privately paid medical services and to study their relationship with the broader primary care workforce.

Future work should incorporate mixed-methods approaches, including physician and clinic surveys, sampling frames that account for multiple manufacturers, and where possible, direct data verification. These methods are needed to better understand practice volume, time allocation, workforce trends, and providers’ motivations for entering the field of aesthetic medicine, all questions that cannot be answered through website based scans alone. However, given typically low response rates in physician surveys, this scan still offers a valuable starting point for understanding this area of practice [21].

## Conclusion

In summary, this study offers a conservative estimate of the number and characteristics of clinics and physicians that publicly advertise aesthetic services in BC. While family medicine was the most represented specialty group, further research is needed to understand how aesthetic medicine fits into their broader clinical practices. As the sector continues to grow, improved data collection and dedicated workforce research will be essential to understanding how these services intersect within Canada’s evolving primary care landscape.

## Data Availability

The data used in this study were derived from publicly available sources, including the Allergan Aesthetics clinic locator (https://locator.allerganaesthetics.ca/) and the public registrant directory of the College of Physicians and Surgeons of British Columbia (https://www.cpsbc.ca/public/registrant-directory). The dataset generated and/or analyzed during the study are available from the corresponding author upon reasonable request.

## Abbreviations

BC: British Columbia
CIHI: Canadian Institute for Health Information
CPSBC: College of Physicians and Surgeons of British Columbia
FP: Family physician
IMG: International medical graduate
LPN: Licensed practical nurse
MD: Doctor of Medicine
NP: Nurse practitioner
RN: Registered nurse
SD: Standard deviation
US: United States
